# Factors related to the resignation and migration of physicians in public health administration agencies using nationwide survey data in Japan

**DOI:** 10.1186/s12913-023-10085-7

**Published:** 2023-10-24

**Authors:** Yasuaki Saijo, Eiji Yoshioka, Yukihiro Sato, Yuki Kunori

**Affiliations:** 1 Division of Public Health and Epidemiology, Department of Social Medicine, Asahikawa Medical University, Asahikawa, Japan; 2 Division of Public Health and Epidemiology, Department of Social Medicine, Asahikawa Medical University, Midorigaoka-higashi 2-1-1-1 Asahikawa, 078-8510 Hokkaido, Japan

**Keywords:** Internal medicine doctor, Rural medicine, Primary care, Board certification, Subspecialty

## Abstract

**Background:**

Physicians in public health administration agencies (public health physicians: PHP) play important roles in public health; however, there are not enough such physicians in Japan. This study aimed to elucidate the factors related to the resignation and migration of PHPs using nationwide survey data.

**Methods:**

Data from the Survey of Physicians, Dentists, and Pharmacists (2010, 2012, 2014, and 2016) were analyzed. The outcome was the resignation of PHPs or migration to public health administration agencies. The explanatory variables in the resignation analysis were age, sex, workplace, and board certification status. The type of work was added as an explanatory variable in the migration analysis, and clinical specialty was added to the clinical doctor-restricted analysis. The odds ratios (ORs) of the explanatory variables were calculated using generalized estimation equations.

**Results:**

In the resignation analysis among PHPs, women had a significantly lower OR, whereas younger PHPs and those with board certifications had significantly higher ORs. In the migration to public health administration agencies analysis among medical doctors, women and those aged between 35 and 39 years had significantly higher ORs, but those with board certifications had significantly lower ORs. Hospital/clinic founders or directors had significantly lower ORs, but the clinic staff and ‘others/not working’ had significantly higher ORs. In the migration to public health administration agencies analysis among clinical physicians, those aged between 35 and 39 years had significantly higher ORs. Still, those with two or more board certifications had significantly lower ORs. Hospital/clinic founders or directors had significantly lower ORs, but the clinic staff had significantly higher ORs. Clinical doctors specializing in surgery and other specialties had significantly lower ORs, but those specializing in pediatrics and psychiatry/psychosomatic medicine had significantly higher ORs.

**Conclusions:**

Having board certifications were significantly related to the resignation of PHPs and migration to public health administration agencies. Women migrated to public health administration agencies more than men and younger PHPs were more likely to resign. However, medical doctors aged between 35 and 39 years were more likely to migrate to public health administration agencies. Similarly, clinic staff, non-clinical physicians, and those whose specialties were pediatrics and psychiatry/psychosomatic medicine were more likely to migrate to public health administration agencies.

**Supplementary Information:**

The online version contains supplementary material available at 10.1186/s12913-023-10085-7.

## Background

In Japan, physicians in public health administration agencies (public health physicians: PHP), including public health centers and public health departments in the local or national government, play important roles in public health [1]. Public health centers operate under the Ministry of Health, Labour and Welfare (MHLW) of Japan, and they perform a wide range of services, such as those involving maternal health, mental health, environmental health, etc. They also play an important role in infection control. The anti-tuberculosis measure was one of their main works because tuberculosis was a leading cause of death in the late 1940s, and intensive measures are still being performed [2, 3]. Furthermore, public health centers and the government as a control tower had crucial roles in the COVID-19 response as they played the joint role of an administrative center for health crisis management [4].

Public health administration agencies in Japan have significant functions, and the directors of public health centers are limited to medical doctors. However, public health centers can hire non-medical doctors with certain qualifications as directors because it is difficult to recruit qualified medical doctors [2, 5]. The number of medical doctors in public health centers declined from 1,239 to 1989 to 730 in 2017 [5]. The MHLW and related societies have been promoting activities to secure enough public health doctors; however, a review insisted that they had not been able to achieve satisfactory results [5].

Thus, because there are not enough PHPs in Japan, the factors affecting their resignation and migration need to be known. Only one nationwide study has been conducted, which reported that younger people tend to resign more, and many come from hospitals [1]. The study used data from 1994 to 2004; however, after compulsory postgraduate clinical training was introduced in 2006, the number of medical doctors going to rural areas or basic medical sciences decreased [6]. Further, multivariable adjustment was not conducted in the study, and no other studies have reported factors that significantly relate to PHP resignation and migration. The purpose of this study was to elucidate the factors related to the resignation and migration of PHPs using nationwide survey data from 2010 to 2016 in Japan.

## Methods

Data from the Survey of Physicians, Dentists, and Pharmacists (2010, 2012, 2014, and 2016), a biennial national census conducted by the Japanese MHLW, were used. All licensed physicians, dentists, and pharmacists in Japan are obligated to register for the survey based on the Medical Practitioners Act. Permission to use the datasets from the MHLW was obtained (Statistics Law, Article 33), and this study was approved by the Asahikawa Medical University Research Ethics Committee (approval number: 21,104 (October 13, 2021)).

**Fig. 1:**
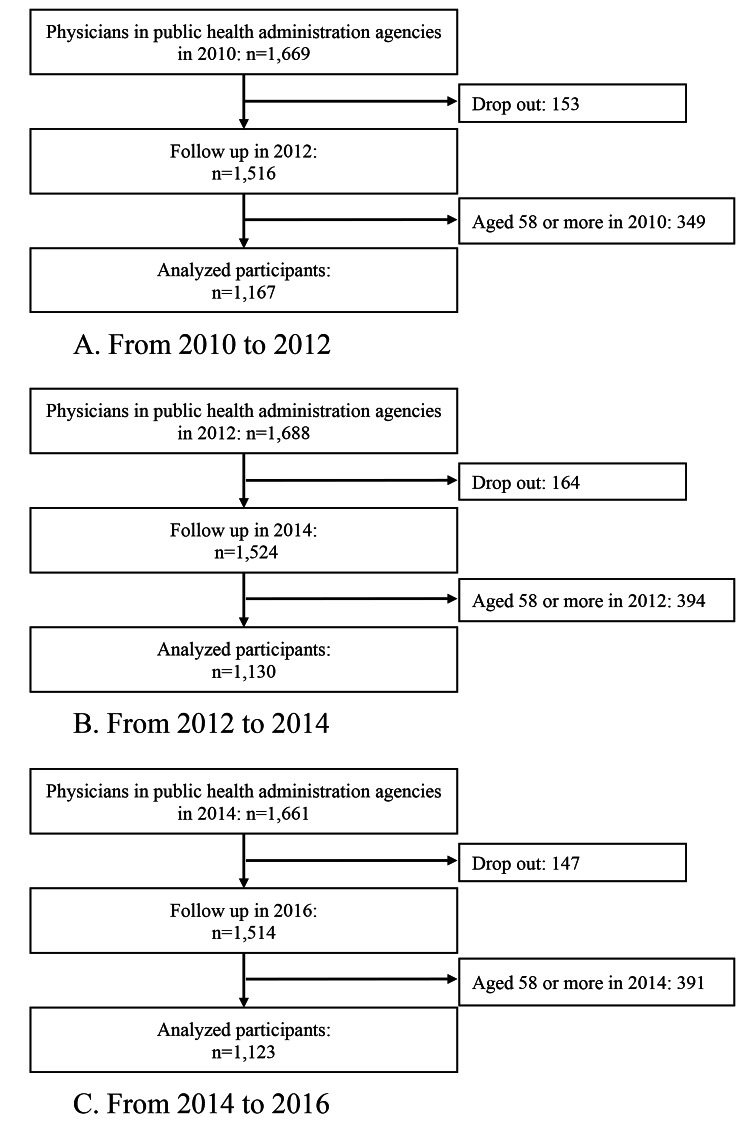
Flow chart of participants regarding the resignation of physicians in public health administration agencies analyses. **A**. From 2010 to 2012. **B**. From 2012 to 2014. **C**. From 2014 to 2016

**Fig. 2:**
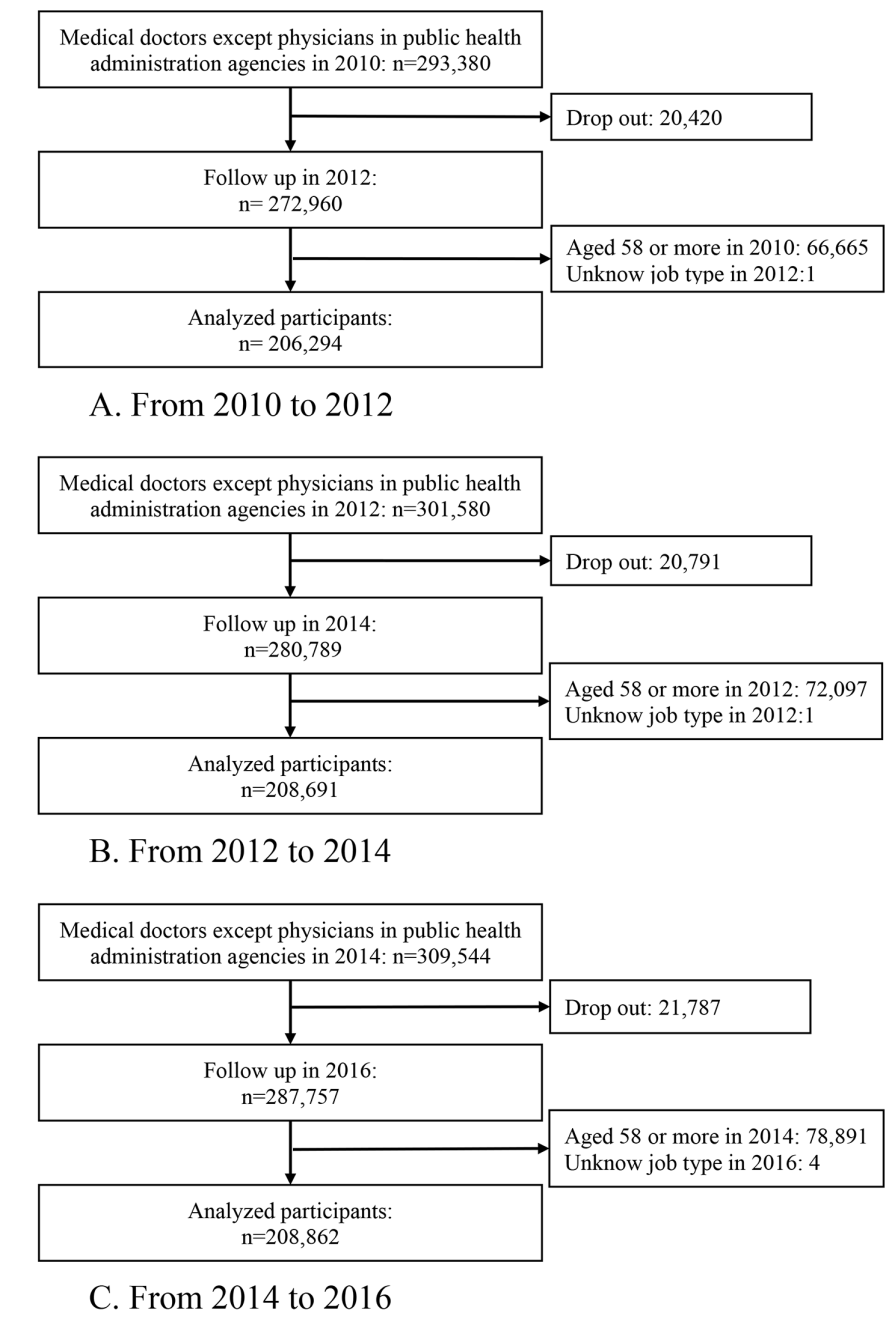
Flow chart of participants regarding the immigration to physicians in public health administration agencies analyses. **A**. From 2010 to 2012. **B**. From 2012 to 2014. **C**. From 2014 to 2016

**Fig. 3:**
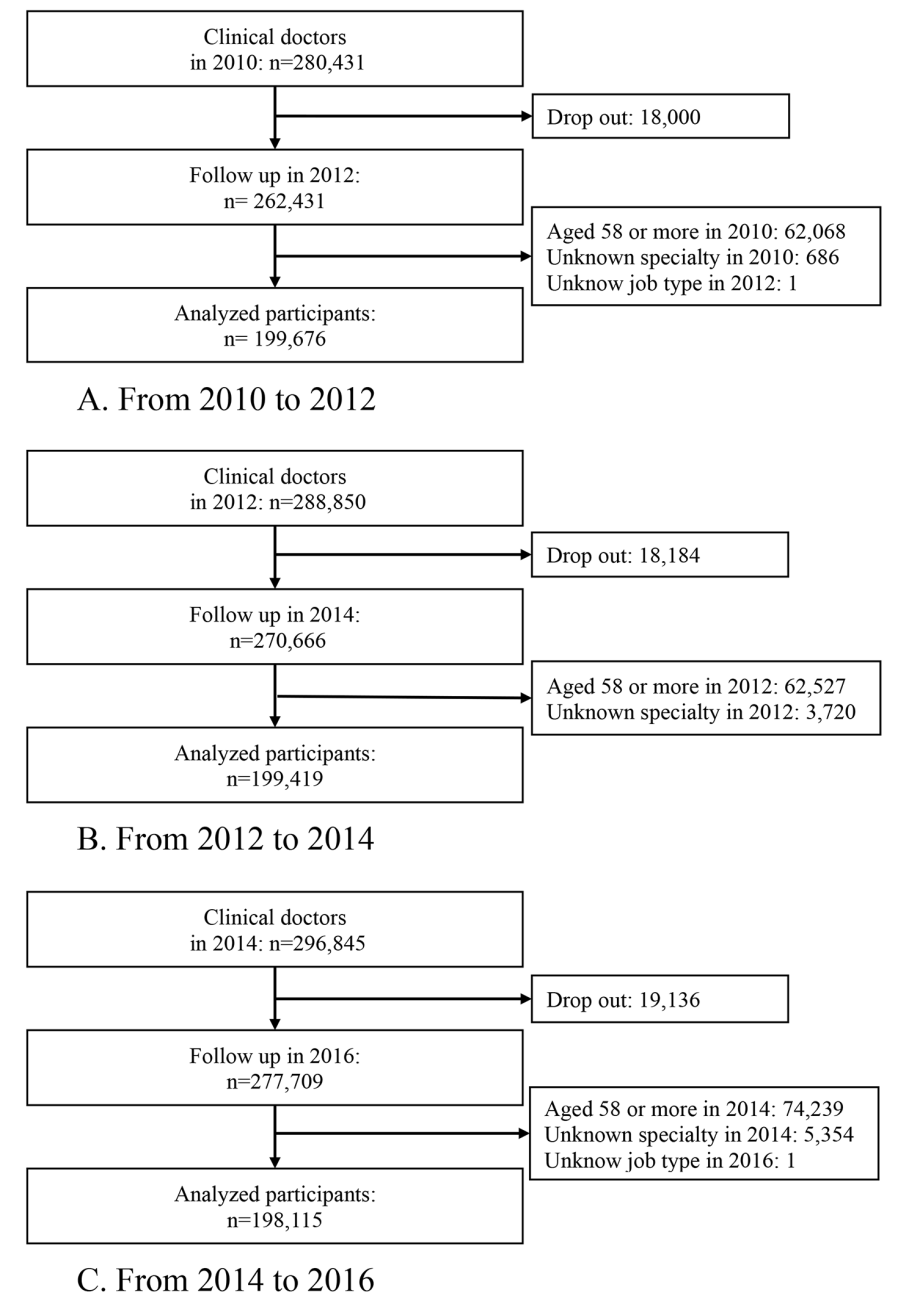
Flow chart of participants regarding the immigration to physicians in public health administration agencies analyses (clinical doctors). **A**. From 2010 to 2012. **B**. From 2012 to 2014. **C**. From 2014 to 2016

The physician registration numbers were used to establish a follow-up dataset, and three types of two-year (2010–2012, 2012–2014, and 2014–2016) follow-up analyses were conducted as follows: (1) odds ratio (OR) of resignation among PHPs (PHPs becoming non-PHPs), (2) OR of migration to public health administration agencies among all medical doctors (non-PHPs becoming PHPs), and (3) OR of migration to public health administration agencies among all clinical physicians (non-PHPs (clinical) becoming PHPs). PHPs were defined as physicians who answered that they worked at administrative agencies such as national and prefectural governments, municipalities, and public health centers. In all clinical physicians’ analyses, the analyzed physicians were restricted to those who answered working at hospitals, clinics, and medical schools (excluding non-clinical work). The flowcharts of the analyses of study participants 1), 2), and 3) are shown in Figs. 1 and 2, and 3, respectively. As the usual retirement age in the public sector was 60 years during the survey period, the age of the participants at baseline was restricted to 57 years or less. The number of participants analyzed in the resignations from public health administration agencies from 2010 to 2012, 2012 to 2014, and 2014 to 2016 were 1,167, 1,130, and 1,123, respectively. The numbers of participants in the immigration to public health administration agencies analysis from 2010 to 2012, 2012 to 2014, and 2014 to 2016 were 206,294, 208,691, and 208,862, respectively. The numbers of participants in the immigration to public health administration agencies analysis (restricted to clinical doctors) from 2010 to 2012, 2012 to 2014, and 2014 to 2016 were 199,679, 199,419, and 198,115, respectively.

The outcomes were resignation from or migration to public health administration agencies. The explanatory variables of the analysis of PHP resignations were age, sex, workplace, and board certification status. Age was categorized as < 29, 30–34, 35–39, 40–44, 45–49, 50–54, and 55–57 years. Workplaces were categorized as large cities, small cities, towns, or villages. The large cities included special wards of Tokyo (Tokubetsu-ku, n = 23), government ordinance cities (Seirei-shi, n = 20), core cities (Chukaku-shi, n = 45), and cities located in the prefectural government (n = 47, overlapping with 1 Tokubetsu-ku, 15 Seirei-shi, and 20 Chukaku-shi). Cities not defined as large were classified as small. The remainders were towns and villages. Because some municipalities were merged and some towns and villages were converted to cities during the follow-up period, the rurality definitions were based on the 2015 census, as it was the only census data during the period (the censuses were surveyed every 5 years). Board certifications were categorized as none, one, two, or more. We used the types of boards asked in the 2010 and 2012 surveys (Supplemental Table 1). As certified psychiatrists have been interviewed since 2014, and the Japan Board of Public Health and Social Medicine started in 2017, they were not included in the analysis.

In the migration to public health administration agencies analyses, the explanatory variables were the abovementioned variables plus the type of work. Types of work were classified as hospital/clinic founder or director, hospital staff, clinic staff, medical school workers (clinical faculty member, clinical staff, or PhD students (clinical)), or others/not working. In the other migration analyses restricted to clinical doctors, after excluding the ‘others/not working’ physicians from the ‘type of work’ variable, the clinical specialty was added as an explanatory variable. The clinical specialty was classified as internal medicine, surgery, pediatrics, obstetrics/gynecology, psychiatry/psychosomatic medicine, other specialties, or junior residents (Supplemental Table 2).

### Statistical analysis

For data with three measurements (2010, 2012, and 2014) of time-varying variables (sex, age, workplace, number of board certifications, type of work, and specialty) and the outcomes (resignation and migration) reported after two years (2012, 2014, and 2016), the random-effects logistic regression generalized estimating equation (GEE) with robust standard errors was used. The GEE considers repeated measures to estimate adjusted ORs for resignation or migration in public health administration agencies. In the sensitivity analyses, each OR of the 2-year follow-up (2010–2012, 2012–2014, 2014–2016) was estimated using the usual multivariable logistic regression analysis. Statistical significance was set at P < 0.05. All analyses were performed using Stata statistical software version 17.0 for Windows (StataCorp, College Station, TX, USA).

## Results

Table 1 lists the number of PHPs per year. Approximately 0.53–0.57% of the registered doctors were PHPs.

**Table 1:** Number of physicians in public health administration agencies in 2010, 2012, 2014, and 2016 among the total registered medical doctors

	2010		2012		2014		2016	
	(n = 295,049)		(N = 303,268)		(N = 311,205)		(N = 319,479)	
	N	%	N	%	N	%	N	%
Physicians in public health administration agencies	1,669	0.57	1,688	0.56	1,661	0.53	1,740	0.54

The characteristics of PHPs in the resignation analysis are shown in Table 2, and approximately 18.2–22.6% resigned.

**Table 2:** Characteristics of physicians in public health administration agencies in the resignation analyses

	2010–2012			2012–2014			2014–2016	
	(N = 1,167)			(N = 1,130)			(N = 1,123)	
	N	%		N	%		N	%
Women	387	33.2		391	34.6		388	34.6
Age (year)								
-29	17	1.5		22	2.0		31	2.8
30–34	89	7.6		95	8.4		75	6.7
35–39	145	12.4		152	13.5		166	14.8
40–44	198	17.0		181	16.0		177	15.8
45–49	253	21.7		220	19.5		220	19.6
50–54	298	25.5		285	25.2		294	26.2
55–57	167	14.3		175	15.5		160	14.3
Workplace								
Large cities	823	70.5		803	71.1		828	73.7
Small cities	308	26.4		299	26.5		268	23.9
Towns or villages	36	3.1		28	2.5		27	2.4
Number of board certifications								
0	895	76.7		833	73.7		731	65.1
1	191	16.4		208	18.4		313	27.9
2 or more	81	6.9		89	7.9		79	7.0
Resignation 2 years later	212 ^*^	18.2		252 ^**^	22.3		254	22.6

^*^ Of 212, 117 were registered as PHP in 2014, and 176 were registered as PHP in 2016. ^**^ Of 252, 99 were registered as PHP in 2016.

The characteristics of medical doctors in the migration to public health administration agencies analysis are shown in Table 3, and approximately 0.1–0.2% migrated.

**Table 3:** Characteristics of medical doctors regarding the migration to physicians in public health administration agencies analyses

	2010–2012			2012–2014			2014–2016	
	(N = 206,294)			(N = 208,691)			(N = 208,862)	
	N	%		N	%		N	%
Women	43,830	21.3		46,864	22.5		49,545	23.7
Age (year)								
-29	25,073	12.2		25,225	12.1		25,205	12.1
30–34	30,147	14.6		30,643	14.7		30,689	14.7
35–39	31,636	15.3		31,386	15.0		31,044	14.9
40–44	32,656	15.8		33,375	16.0		33,310	16.0
45–49	34,891	16.9		33,846	16.2		33,470	16.0
50–54	34,313	16.6		34,763	16.7		34,662	16.6
55–57	17,578	8.5		19,453	9.3		20,482	9.8
Workplace								
Large cities	119,160	57.8		121,390	58.2		122,808	58.8
Small cities	76,776	37.2		77,323	37.1		76,292	36.5
Towns or villages	10,358	5.0		9,978	4.8		9,762	4.7
Number of board certifications								
0	99,656	48.3		96,461	46.2		88,433	42.3
1	69,844	33.9		74,045	35.5		80,356	38.5
2 or more	36,794	17.8		38,185	18.3		40,073	19.2
Type of work								
Hospital/ clinic founder or director	35,481	17.2		33,046	15.8		30,639	14.7
Hospital staff	104,245	50.5		107,078	51.3		108,260	51.8
Clinic staff	16,420	8.0		17,119	8.2		17,407	8.3
Medical school*	44,216	21.4		45,896	22.0		47,163	22.6
Others/not working	5,932	2.9		5,552	2.7		5,393	2.6
Migration to public health administration agencies 2 years later	280	0.1		323	0.2		346	0.2

*Clinical faculty members, clinical staff, or PhD students (clinical students)

The characteristics of clinical doctors migrating to public health administration agencies are shown in Table 4, and approximately 0.1% migrated.

**Table 4:** Characteristics of clinical doctors regarding the migration to public health administration agencies analyses

	2010–2012			2012–2014			2014–2016	
	(N = 199,676)			(N = 199,419)			(N = 198,115)	
	N	%		N	%		N	%
Women	42,248	21.2		45,066	22.6		47,641	24.1
Age (year)								
-29	24,777	12.4		24,800	12.4		24,713	12.5
30–34	29,349	14.7		29,518	14.8		29,219	14.8
35–39	30,719	15.4		30,037	15.1		29,469	14.9
40–44	31,600	15.8		31,812	16.0		31,524	15.9
45–49	33,547	16.8		32,102	16.1		31,556	15.9
50–54	32,815	16.4		32,786	16.4		32,510	16.4
55–57	16,869	8.5		18,364	9.2		19,124	9.7
Workplace								
Large cities	114,787	57.5		115,517	57.9		116,030	58.6
Small cities	74,880	37.5		74,284	37.3		72,782	36.7
Towns or villages	10,009	5.0		9,618	4.8		9,303	4.7
Number of board certifications								
0	95,277	47.7		91,786	46.0		83,782	42.3
1	68,389	34.3		70,645	35.4		75,755	38.2
2 or more	36,010	18.0		36,988	18.6		38,578	19.5
Type of work								
Hospital/ clinic founder or director	35,325	17.7		32,783	16.4		30,359	15.3
Hospital staff	103,907	52.0		104,686	52.5		104,759	52.9
Clinic staff	16,349	8.2		16,960	8.5		17,230	8.7
Medical school*	44,095	22.1		44,990	22.6		45,767	23.1
Specialty								
Internal medicine	68,126	34.1		68,522	34.4		68,266	34.5
Surgery	42,223	21.2		39,393	19.8		37,023	18.7
Pediatrics	10,873	5.5		11,045	5.5		10,945	5.5
Obstetrics/gynecology	7,843	3.9		8,198	4.1		8,300	4.2
Psychiatry/psychosomatic medicine	10,553	5.3		10,686	5.4		10,723	5.4
Other specialties	46,174	23.1		47,159	23.7		48,248	24.4
Junior resident	13,884	7.0		14,416	7.2		14,610	7.4
Migration to public health administration agencies 2 years later	226	0.1		263	0.1		277	0.1

*Clinical faculty members, clinical staff, or PhD students (clinical students)

The ORs for resignation among PHPs in the GEE analysis are presented in Table 5. Women had a significantly lower OR (OR = 0.58, 95% confidence interval (CI): 0.41–0.82), and younger PHPs had significantly higher ORs (≤ 29 years: OR = 4.33, 95%CI: 1.33–14.03; 30–34 years: OR = 4.39, 95%CI: 2.25–8.53). Moreover, those with board certifications had significantly higher ORs (one certification: OR = 1.59, 95%CI: 1.06–2.39; two or more certifications: OR = 2.98, 95%CI: 1.79–4.95). The workplace was not a significant factor. In the sensitivity analyses, each OR of the 2-year follow-up (2010–2012, 2012–2014, 2014–2016) had similar results (Supplemental Table 3).

**Table 5:** Odds ratios for the resignation of physicians in public health administration agencies 2 years later (N = 1,861, 2-year observation = 3,420)

	OR	95%CI			P
Women (vs. men)	0.58	0.41	—	0.82	0.002
Age (year)					
-29	4.33	1.33	—	14.03	0.015
30–34	4.39	2.25	—	8.55	< 0.001
35–39	2.65	1.42	—	4.93	0.002
40–44	1.72	0.94	—	3.16	0.079
45–49	1.56	0.87	—	2.81	0.134
50–54	1.11	0.61	—	2.02	0.724
55–57	1.00				
Workplace					
Large cities	1.00				
Small cities	0.71	0.48	—	1.03	0.073
Towns or villages	0.59	0.20	—	1.71	0.331
Number of board certifications			—		
0	1.00				
1	1.59	1.06	—	2.39	0.024
2 or more	2.98	1.79	—	4.95	< 0.001

The ORs for migration to public health administration agencies among medical doctors in the GEE analysis are shown in Table 6. Women had a significantly higher OR (OR = 1.23, 95%CI: 1.04–1.44), and medical doctors aged between 35 and 39 years had significantly higher ORs (OR = 1.42, 95%CI: 1.04–1.93). Moreover, those with board certifications had significantly lower ORs (one certification: OR = 0.64, 95%CI: 0.54–0.76; two or more certifications: OR = 0.53, 95%CI: 0.41–0.67). Hospital/clinic founders or directors had significantly lower ORs (OR = 0.11, 95%CI: 0.06–0.19); however, clinic staff and ‘others/not working’ had significantly higher ORs (clinic staff: OR = 1.80, 95%CI: 1.43–2.27; ‘others/not working’: OR = 8.49, 95%CI: 6.68–10.79). Similarly, the workplace was not a significant factor. In the sensitivity analyses (Supplemental Table 4), board certification and type of work’s ORs of the 2-year follow-up (2010–2012, 2012–2014, 2014–2016) had almost similar results. However, significant results regarding women were obtained only in the 2010–2012 analysis, and significant results regarding those aged between 35 and 39 years were obtained only in the 2014–2016 analysis. In contrast, small cities, towns, or villages in the 2014–2016 analysis had significantly lower ORs.

**Table 6:** Odds ratios for migration to physicians in public health administration agencies 2 years later among medical doctors (N = 251,674, 2-year observation = 623,847)

	OR	95%CI			P
Women	1.23	1.04	—	1.44	0.014
Age (year)					
-29	0.75	0.52	—	1.08	0.116
30–34	1.16	0.83	—	1.60	0.381
35–39	1.42	1.04	—	1.93	0.026
40–44	1.03	0.75	—	1.42	0.865
45–49	1.06	0.77	—	1.47	0.701
50–54	0.82	0.59	—	1.14	0.237
55–57	1.00				
Workplace					
Large cities	1.00				
Small cities	0.91	0.78	—	1.06	0.212
Towns or villages	0.81	0.57	—	1.15	0.238
Number of board certifications					
0	1.00				
1	0.64	0.54	—	0.76	< 0.001
2 or more	0.53	0.41	—	0.67	< 0.001
Type of work					
Hospital/ clinic founder or director	0.11	0.06	—	0.19	< 0.001
Hospital staff	1.00				
Clinic staff	1.80	1.43	—	2.27	< 0.001
Medical school*	1.10	0.91	—	1.32	0.319
Others/not working	8.49	6.68	—	10.79	< 0.001

*Clinical faculty members, clinical staff, or PhD students (clinical students)

The ORs for migration to public health administration agencies among clinical doctors in the GEE analysis are shown in Table 7. Women and workplaces showed no statistical significance. Clinical doctors aged between 35 and 39 years had significantly higher ORs (OR = 1.50, 95%CI: 1.05–2.14), and those with two or more board certifications had significantly lower ORs (OR = 0.75, 95%CI: 0.57–0.99). Hospital/clinic founders or directors had significantly lower ORs (OR = 0.11, 95%CI: 0.06–0.19), but clinic staff had significantly higher ORs (OR = 1.93, 95%CI: 1.52–2.45). Clinical doctors whose specialty was surgery had significantly lower ORs (OR = 0.69, 95%CI: 0.54–0.89), and those in other specialties also had significantly lower ORs (OR = 0.69, 95%CI: 0.54–2.11). However, those specializing in pediatrics had significantly higher ORs (OR = 1.54, 95%CI: 1.13–2.11), and those in psychiatry/psychosomatic medicine also had significantly higher ORs (OR = 3.90, 95%CI: 3.00–5.08). In the sensitivity analyses (Supplemental Table 5), the type of work’s ORs of the 2-year follow-up (2010–2012, 2012–2014, 2014–2016) had almost similar results. Age and specialty had similar tendencies, but significant results were obtained in the 2010–2012 analysis for those aged between 35 and 39 years, those in other specialties, and those in surgery and pediatrics disappeared. Moreover, significant results regarding women were obtained only in the 2010–2012 analysis, and those regarding two or more board certifications were obtained in the 2012–2014 analysis. The small cities, towns, and villages in the 2014–2016 analysis had significantly lower ORs.

**Table 7:** Odds ratios for migration to physicians in public health administration agencies 2 years later among clinical doctors (N = 246,144, 2-year observation = 597,210)

	OR	95%CI			P
Women	1.14	0.96	—	1.37	0.142
Age (year)					
-29	1.03	0.67	—	1.59	0.893
30–34	1.41	0.97	—	2.06	0.072
35–39	1.50	1.05	—	2.14	0.025
40–44	1.02	0.70	—	1.48	0.924
45–49	1.08	0.74	—	1.56	0.701
50–54	0.82	0.56	—	1.22	0.331
55–57	1.00				
Workplace					
Large cities	1.00				
Small cities	0.87	0.74	—	1.03	0.112
Towns or villages	0.78	0.53	—	1.16	0.221
Number of board certifications					
0	1.00				
1	0.89	0.72	—	1.09	0.264
2 or more	0.75	0.57	—	0.99	0.044
Type of work					
Hospital/ clinic founder or director	0.11	0.06	—	0.19	< 0.001
Hospital staff	1.00				
Clinic staff	1.93	1.52	—	2.45	< 0.001
Medical school*	1.13	0.94	—	1.37	0.202
Specialty					
Internal medicine	1.00				
Surgery	0.69	0.54	—	0.89	0.005
Pediatrics	1.54	1.13	—	2.11	0.006
Obstetrics/gynecology	0.65	0.40	—	1.04	0.069
Psychiatry/psychosomatic medicine	3.90	3.00	—	5.08	< 0.001
Other specialties	0.69	0.54	—	0.87	0.002
Junior resident	0.98	0.70	—	1.38	0.907

*Clinical faculty members, clinical staff, or PhD students (clinical students)

## Discussion

In this longitudinal national survey data analysis of Japanese medical doctors, we found that women tended to continue in public health administration agencies and that lower age and board certifications were negative factors for public health administration agency retention. In the medical doctors’ analysis, we found that women and medical doctors aged between 35 and 39 years tended to migrate to public health administration agencies and that having board certifications was a negative factor. Regarding job type, hospital/clinic founders or directors tended to continue their work, but clinic staff and ‘others/not working’ tended to migrate to public health administration agencies. Regarding the specialties in clinical doctors’ analysis, those who specialized in surgery did not tend to migrate to public health administration agencies, but those who specialized in pediatrics or psychiatry/psychosomatic medicine did. To the best of our knowledge, this is the first study to report the factors for the retention and migration in public health administration agencies among medical doctors using multivariable-adjusted analysis of Japanese nationwide survey data.

Board certification was significantly associated with resigning from and migrating to a public health administration agency. Having board certification implies that they have specific clinical experience and can be easily accepted in clinical areas. The quality of board certification during the study was not validated, and the certifications tended to be easy to obtain and maintain [7]. However, the new board certification training system started in 2018 to coordinate and harmonize the criteria, and the process was led by the Panel on Board Certification within the Japanese MHLW [8]. Thus, as the new board certifications are more difficult to keep compared to the former ones and the PHPs who do not do clinical work find it harder to keep them, it may accelerate the avoidance of public health administration agency work among the new board-certified younger physicians. There are variations in public health administration agency work across countries [9, 10], and some in Korea involve basic disease treatment in primary care settings and emergency medicine [11]. Therefore, adding clinical work like that in Korea to public health administration agencies in Japan may aid the retention of workers, especially for those with board certifications.

Hospital/clinic founders or directors tended to continue their work in our study, which was plausible because they funded their clinics or took positions by gaining more clinical experience. The clinic staff migrated more than the hospital staff. Clinical staff mainly provide primary care, which is closely related to public health [12]. These similarities may facilitate the migration of clinical staff. ‘Others/not working’ also had significantly higher ORs and non-clinician medical doctors were possible workers in public health administration agencies.

Pediatrics and psychiatry are highly represented in public health administrative agencies and have a relatively close relationship with public health [1]. This could explain the higher ORs in pediatrics and psychiatry/psychosomatic medicine. In contrast, surgery and other specialties had significantly lower ORs, which may be because surgery requires long-term training in technique, and other specialties that include relatively finely divided areas are not very close to public health and preventive medicine.

Women resigned less in public health administrative agencies compared to men, and they migrated more to public health administrative agencies in the medical doctors’ analysis in our study. In Japan, female physicians, who are expected to have babies or to raise children, agonize between their identities as “women” and “doctors” and are troubled by the female stereotype that “child-rearing is a woman’s job” [13, 14]. Female physicians choose specializations such as family practice and pediatrics but are less likely to work excessive hours and are more likely to include more preventive care in their practice [15]. Although the COVID-19 pandemic put a great burden on public health officials, a PHP’s work, with no emergency patients or night shifts, might preferentially select for female physicians during the study period. This is because the stereotypical gender role of “child rearing is a woman’s job” pertains to Japanese physicians [16].

The younger the PHP, the more likely they resign from work. Since younger people can change jobs more easily than older ones, measures should be implemented to keep younger PHPs back. The peak age stratum of migration was 35–39 years. One of the typical clinical doctors’ goals is to open a new private solo practice after spending more than 10 years working in hospitals [17]. This seemed to reflect the frequent career change periods of Japanese clinical doctors.

There is a worldwide shortage of healthcare workers in rural areas [18], and the retention of physicians is low in rural areas in Japan [19]. As the public health centers in rural areas are mainly prefectural-level centers [5] and prefectural personnel is transferred regularly, they are promised to be moved to non-rural areas in a few years. Therefore, quitting as a PHP to avoid rural work is less likely to occur.

There is a large variety of public health work across countries [20]. The educational system and jobs of PHPs differ between the United States and Canada [21], and PHPs in Korea have some clinical work, in contrast to Japanese PHPs [11]. The results of our study cannot be directly applied to other countries; however, physicians’ clinical careers, especially board certifications, should be considered for PHP recruitment.

This study had several limitations. First, there are several types of administrative agencies, such as national and prefectural governments, municipalities, and public health centers, which can be categorized as cities, special wards, and prefectural-level centers [5], but these factors were not taken into consideration in the analysis because detailed data were unavailable in the survey data. Second, the PHP dropout rate ranged from 9.2 to 9.8% (Fig. 1), while that of physicians ranged from 6.9 to 7.0% (Fig. 2). The reason for the slightly higher PHP dropout rates is unknown; however, it possible that because public health centers are included as registration sites for the Survey of Physicians, Dentists, and Pharmacists, the incentive for registration among PHP was higher. However, after reignition, the incentive may have diminished, which could have potentially led to an underestimation of PHP dropout rates. Third, in the resignation analyses, a relatively large number of resigned PHPs were re-registered as PHPs after two or four years. This may be because it is easier for them to return to work in a profession in which they have experience. Furthermore, there may be some transfers to people other than PHP who work as municipal employees, and the transfers of municipal employees were not considered in this study. Fourth, public health centers have a COVID-19 burden and usually change to cope with new pandemics and health crises [22]. Changes in work modalities after the COVID-19 pandemic were not considered. Finally, the sensitivity analyses of each OR period showed some discrepancies. This could be because of the smaller sample sizes compared with the GEE analyses; however, the disappearance of the significant results regarding the women’s ORs in later periods may reflect changes over time.

## Conclusion

Having board certifications was a negative factor for PHP retention and migration. Moreover, women continued to migrate to public health administration agencies more than men and younger PHPs were more likely to resign. However, medical doctors aged between 35 and 39 years were more likely to migrate to public health administration agencies. Similarly, the clinic staff, non-clinical physicians, and those whose specialties were pediatrics and psychiatry/psychosomatic medicine were more likely to migrate to public health administration agencies. Making PHPs maintain their clinical board certification is a possible countermeasure, and it is necessary to make public health administration agencies attractive to younger PHPs. Individuals who can migrate to public health administration agencies include those aged between 35 and 39 years, clinic staff, non-clinical medical doctors, and those specializing in pediatrics and psychiatry/psychosomatic medicine.

### Electronic supplementary material

Below is the link to the electronic supplementary material.

Supplementary Material 1

Supplementary Material 2

Supplementary Material 3

Supplementary Material 4

Supplementary Material 5

## Data Availability

The raw data collected for government statistics were not shared because of restrictions stipulated by the MHLW. Data are available from the MLWL for researchers who meet the criteria for access to confidential data. Contact information is as follow: Phone + 81-166-68-2401, email y-saijo@asahikawa.med.ac.jp.

## Abbreviations

PHP: public health physician
OR: odds ratio
GEE: generalized estimating equation
CI: confidence interval
MHLW: Ministry of Health, Labour and Welfare
