# Supplementary material for: Factors related to the resignation and migration of physicians in public health administration agencies using nationwide survey data in Japan
Source: BMC Health Serv Res. 2023 Oct 24;23:1143. doi: 10.1186/s12913-023-10085-7 (PMC10599074; doi:10.1186/s12913-023-10085-7)
Supplement: Supplementary file 1 — Supplementary Material 1 [file 12913_2023_10085_MOESM1_ESM.docx]

Supplemental Table 1. List of board certifications

| Internal medicine |
| --- |
| Respiratory medicine |
| Cardiology |
| Gastroenterology |
| Nephrology |
| Hepatology |
| Nephrology |
| Diabetology |
| Endocrinology |
| Hematology |
| Dermatology |
| Allergology |
| Rheumatology |
| Infectious diseases |
| Pediatrics |
| Psychosomatic medicine |
| Surgery |
| Respiratory surgery |
| Cardiovascular surgery |
| Breast oncology |
| Broncho-esophagology |
| Gastrointestinal Surgery |
| Urology |
| Neurosurgery |
| Orthopedics |
| Plastic surgery |
| Ophthalmology |
| Otolaryngology |
| Pediatric surgery |
| Obstetrics and Gynecology |
| Rehabilitation |
| Radiology |
| Anesthesiology |
| Pathology |
| Emergency medicine |
| Ultrasonics in medicine |
| Clinical cytology |
| Dialysis therapy |
| Gerontology |
| Gastroenterological endoscopy |
| Medical genetics |
| Kampo medicine |
| Laser medicine |
| Respiratory endoscopy |
| Nuclear medicine |
| Coloproctology |
| Gynecologic oncology |
| Pain management |
| Burn injuries |
| Neuroendovascular therapy |
| Medical oncology |
| Perinatal and neonatal medicine |
| Reproductive medicine |
| Child neurology |
| Consultation-Liaison psychiatrist |
