# Supplementary material for: Factors related to the resignation and migration of physicians in public health administration agencies using nationwide survey data in Japan
Source: BMC Health Serv Res. 2023 Oct 24;23:1143. doi: 10.1186/s12913-023-10085-7 (PMC10599074; doi:10.1186/s12913-023-10085-7)
Supplement: Supplementary file 2 — Supplementary Material 2 [file 12913_2023_10085_MOESM2_ESM.docx]

Supplemental Table 2. Classification of specialties

| Category | Specialty |
| --- | --- |
| Internal medicine | Internal medicine |
|  | Respiratory medicine |
|  | Cardiovascular medicine |
|  | Gastrointestinal medicine |
|  | Nephrology |
|  | Nephrology |
|  | Diabetology |
|  | Hematology |
|  | Infectious diseases |
|  | General |
| Surgery | Surgery |
|  | Respiratory surgery |
|  | Cardiovascular surgery |
|  | Breast surgery |
|  | Trachea esophagus surgery |
|  | Gastrointestinal Surgery |
|  | Anal surgery |
|  | Neurosurgery |
|  | Orthopedics |
|  | Plastic surgery |
|  | Pediatric surgery |
| Pediatrics | Pediatrics |
| Obstetrics/ gynecology | Obstetrics/gynecology |
|  | Obstetrics |
|  | Gynecology |
| Psychiatry/psychosomatic medicine | Psychiatry |
|  | Psychosomatic medicine |
| Other specialties | Dermatology |
|  | Allergology |
|  | Rheumatology |
|  | Urology |
|  | Cosmetic Surgery |
|  | Ophthalmology |
|  | Otolaryngology |
|  | Rehabilitation |
|  | Radiology |
|  | Anesthesiology |
|  | Diagnostic Pathology |
|  | Clinical laboratory |
|  | Emergency medicine |
|  | Other specialties |
| Junior resident | Junior resident |
