# Supplementary material for: Factors related to the resignation and migration of physicians in public health administration agencies using nationwide survey data in Japan
Source: BMC Health Serv Res. 2023 Oct 24;23:1143. doi: 10.1186/s12913-023-10085-7 (PMC10599074; doi:10.1186/s12913-023-10085-7)
Supplement: Supplementary file 3 — Supplementary Material 3 [file 12913_2023_10085_MOESM3_ESM.docx]

Supplemental Table 3. Odds ratios for the resignation of physicians in public health administration agencies over 2 years

|  | 2010-2012 | | | | |  | 2012-2014 | | | | |  | 2014-2016 | | | | |
| --- | --- | --- | --- | --- | --- | --- | --- | --- | --- | --- | --- | --- | --- | --- | --- | --- | --- |
|  | (N=1,167) | | | | |  | (N=1,130) | | | | |  | (N=1,123) | | | | |
|  | OR | 95%CI | | | P |  | OR | 95%CI | | | P |  | OR | 95%CI | | | P |
| Women (vs. men) | 0.58 | 0.41 | — | 0.82 | 0.002 |  | 0.56 | 0.40 | — | 0.77 | <0.001 |  | 0.67 | 0.48 | — | 0.92 | 0.014 |
| Age (year) |  |  |  |  |  |  |  |  |  |  |  |  |  |  |  |  |  |
| -29 | 4.33 | 1.33 | — | 14.03 | 0.015 |  | 8.66 | 3.31 | — | 22.67 | <0.001 |  | 13.83 | 5.64 | — | 33.90 | <0.001 |
| 30-34 | 4.39 | 2.25 | — | 8.55 | <0.001 |  | 6.43 | 3.49 | — | 11.83 | <0.001 |  | 8.68 | 4.27 | — | 17.66 | <0.001 |
| 35-39 | 2.65 | 1.42 | — | 4.93 | 0.002 |  | 3.79 | 2.17 | — | 6.64 | <0.001 |  | 5.54 | 2.98 | — | 10.30 | <0.001 |
| 40-44 | 1.72 | 0.94 | — | 3.16 | 0.079 |  | 2.05 | 1.17 | — | 3.59 | 0.013 |  | 2.72 | 1.44 | — | 5.15 | 0.002 |
| 45-49 | 1.56 | 0.87 | — | 2.81 | 0.134 |  | 1.19 | 0.67 | — | 2.09 | 0.550 |  | 2.31 | 1.24 | — | 4.29 | 0.008 |
| 50-54 | 1.11 | 0.61 | — | 2.02 | 0.724 |  | 0.92 | 0.53 | — | 1.61 | 0.772 |  | 1.61 | 0.87 | — | 2.98 | 0.133 |
| 55-57 | 1.00 |  |  |  |  |  | 1.00 |  |  |  |  |  | 1.00 |  | — |  |  |
| Workplace |  |  |  |  |  |  |  |  |  |  |  |  |  |  |  |  |  |
| Large cities | 1.00 |  |  |  |  |  | 1.00 |  |  |  |  |  | 1.00 |  |  |  |  |
| Small cities | 0.71 | 0.48 | — | 1.03 | 0.073 |  | 1.07 | 0.76 | — | 1.52 | 0.695 |  | 0.96 | 0.67 | — | 1.38 | 0.818 |
| Towns or villages | 0.59 | 0.20 | — | 1.71 | 0.331 |  | 1.87 | 0.77 | — | 4.54 | 0.168 |  | 1.72 | 0.68 | — | 4.34 | 0.249 |
| Number of board certifications |  |  |  |  |  |  |  |  | — |  |  |  |  |  |  |  |  |
| 0 | 1.00 |  |  |  |  |  | 1.00 |  |  |  |  |  | 1.00 |  |  |  |  |
| 1 | 1.59 | 1.06 | — | 2.39 | 0.024 |  | 1.96 | 1.35 | — | 2.83 | <0.001 |  | 2.83 | 2.04 | — | 3.93 | <0.001 |
| 2 or more | 2.98 | 1.79 | — | 4.95 | <0.001 |  | 3.02 | 1.84 | — | 4.95 | <0.001 |  | 2.41 | 1.39 | — | 4.20 | 0.002 |
