# Supplementary material for: Factors related to the resignation and migration of physicians in public health administration agencies using nationwide survey data in Japan
Source: BMC Health Serv Res. 2023 Oct 24;23:1143. doi: 10.1186/s12913-023-10085-7 (PMC10599074; doi:10.1186/s12913-023-10085-7)
Supplement: Supplementary file 4 — Supplementary Material 4 [file 12913_2023_10085_MOESM4_ESM.docx]

Supplemental Table 4. Odds ratios for migration to physicians in public health administration agencies 2 years later among medical doctors

|  | 2010-2012 | | | | |  | 2012-2014 | | | | |  | 2014-2016 | | | | | |
| --- | --- | --- | --- | --- | --- | --- | --- | --- | --- | --- | --- | --- | --- | --- | --- | --- | --- | --- |
|  | (N=206,294) | | | | |  | (N=208,691) | | | | |  | (N=208,862) | | | | | |
|  | OR | 95%CI | | | P |  | OR | 95%CI | | | P |  | OR | 95%CI | | | P |  |
| Women | 1.58 | 1.22 | — | 2.04 | 0.001 |  | 1.14 | 0.89 | — | 1.46 | 0.287 |  | 0.97 | 0.76 | — | 1.23 | 0.777 |  |
| Age (year) |  |  |  |  |  |  |  |  |  |  |  |  |  |  |  |  |  |  |
| -29 | 0.76 | 0.42 | — | 1.38 | 0.367 |  | 0.81 | 0.46 | — | 1.44 | 0.480 |  | 0.75 | 0.42 | — | 1.34 | 0.338 |  |
| 30-34 | 0.99 | 0.58 | — | 1.70 | 0.977 |  | 1.03 | 0.61 | — | 1.75 | 0.908 |  | 1.49 | 0.92 | — | 2.43 | 0.107 |  |
| 35-39 | 0.83 | 0.48 | — | 1.43 | 0.496 |  | 1.53 | 0.93 | — | 2.52 | 0.092 |  | 1.92 | 1.20 | — | 3.05 | 0.006 |  |
| 40-44 | 0.81 | 0.47 | — | 1.39 | 0.441 |  | 1.05 | 0.62 | — | 1.76 | 0.860 |  | 1.25 | 0.77 | — | 2.02 | 0.374 |  |
| 45-49 | 1.03 | 0.61 | — | 1.74 | 0.902 |  | 1.23 | 0.74 | — | 2.04 | 0.425 |  | 0.94 | 0.56 | — | 1.56 | 0.808 |  |
| 50-54 | 0.73 | 0.42 | — | 1.28 | 0.272 |  | 0.85 | 0.49 | — | 1.45 | 0.543 |  | 0.93 | 0.56 | — | 1.54 | 0.777 |  |
| 55-57 | 1.00 |  |  |  |  |  | 1.00 |  |  |  |  |  | 1.00 |  |  |  |  |  |
| Workplace |  |  |  |  |  |  |  |  |  |  |  |  |  |  |  |  |  |  |
| Large cities | 1.00 |  |  |  |  |  | 1.00 |  |  |  |  |  | 1.00 |  |  |  |  |  |
| Small cities | 0.95 | 0.73 | — | 1.22 | 0.672 |  | 1.08 | 0.85 | — | 1.36 | 0.548 |  | 0.79 | 0.62 | — | 1.00 | 0.046 |  |
| Towns or villages | 0.89 | 0.51 | — | 1.57 | 0.698 |  | 1.20 | 0.74 | — | 1.96 | 0.454 |  | 0.44 | 0.22 | — | 0.90 | 0.024 |  |
| Number of board certifications |  |  |  |  |  |  |  |  |  |  |  |  |  |  |  |  |  |  |
| 0 | 1.00 |  |  |  |  |  | 1.00 |  |  |  |  |  | 1.00 |  |  |  |  |  |
| 1 | 0.64 | 0.47 | — | 0.86 | 0.003 |  | 0.58 | 0.44 | — | 0.76 | <0.001 |  | 0.73 | 0.57 | — | 0.95 | 0.017 |  |
| 2 or more | 0.64 | 0.43 | — | 0.95 | 0.026 |  | 0.41 | 0.28 | — | 0.62 | <0.001 |  | 0.61 | 0.43 | — | 0.87 | 0.006 |  |
| Type of work |  |  |  |  |  |  |  |  |  |  |  |  |  |  |  |  |  |  |
| Hospital/ clinic founder or director | 0.16 | 0.07 | — | 0.37 | <0.001 |  | 0.07 | 0.02 | — | 0.24 | <0.001 |  | 0.13 | 0.05 | — | 0.32 | <0.001 |  |
| Hospital staff | 1.00 |  |  |  |  |  | 1.00 |  |  |  |  |  | 1.00 |  |  |  |  |  |
| Clinic staff | 1.76 | 1.19 | — | 2.60 | 0.005 |  | 1.86 | 1.32 | — | 2.64 | <0.001 |  | 1.59 | 1.11 | — | 2.27 | 0.011 |  |
| Medical school* | 1.27 | 0.93 | — | 1.73 | 0.128 |  | 1.16 | 0.87 | — | 1.56 | 0.304 |  | 0.90 | 0.68 | — | 1.19 | 0.465 |  |
| Others/not working | 7.09 | 5.04 | — | 9.98 | 0.000 |  | 6.42 | 4.62 | — | 8.92 | 0.000 |  | 7.03 | 5.17 | — | 9.57 | <0.001 |  |

*Clinical faculty members, clinical staff, or PhD students (clinical students).
