# Supplementary material for: Factors related to the resignation and migration of physicians in public health administration agencies using nationwide survey data in Japan
Source: BMC Health Serv Res. 2023 Oct 24;23:1143. doi: 10.1186/s12913-023-10085-7 (PMC10599074; doi:10.1186/s12913-023-10085-7)
Supplement: Supplementary file 5 — Supplementary Material 5 [file 12913_2023_10085_MOESM5_ESM.docx]

Supplemental Table 5. Odds ratios for migration to physicians in public health administration agencies 2 years later among clinical doctors

|  | 2010-2012 | | | | |  | 2012-2014 | | | | |  | 2014-2016 | | | | |
| --- | --- | --- | --- | --- | --- | --- | --- | --- | --- | --- | --- | --- | --- | --- | --- | --- | --- |
|  | (N=199,676) | | | | |  | (N=199,419) | | | | |  | (N=198,115) | | | | |
|  | OR | 95%CI | | | P |  | OR | 95%CI | | | P |  | OR | 95%CI | | | P |
| Women | 1.39 | 1.04 | — | 1.87 | 0.026 |  | 1.10 | 0.84 | — | 1.45 | 0.487 |  | 0.97 | 0.74 | — | 1.27 | 0.812 |
| Age (year) |  |  |  |  |  |  |  |  |  |  |  |  |  |  |  |  |  |
| -29 | 1.04 | 0.51 | — | 2.11 | 0.920 |  | 1.21 | 0.57 | — | 2.54 | 0.622 |  | 0.84 | 0.41 | — | 1.71 | 0.635 |
| 30-34 | 1.11 | 0.60 | — | 2.04 | 0.748 |  | 1.49 | 0.77 | — | 2.88 | 0.231 |  | 1.56 | 0.88 | — | 2.75 | 0.127 |
| 35-39 | 0.81 | 0.44 | — | 1.51 | 0.510 |  | 1.91 | 1.02 | — | 3.58 | 0.044 |  | 1.79 | 1.04 | — | 3.08 | 0.034 |
| 40-44 | 0.74 | 0.40 | — | 1.39 | 0.351 |  | 1.27 | 0.66 | — | 2.44 | 0.472 |  | 1.04 | 0.59 | — | 1.84 | 0.887 |
| 45-49 | 0.94 | 0.51 | — | 1.72 | 0.833 |  | 1.42 | 0.74 | — | 2.71 | 0.292 |  | 0.93 | 0.52 | — | 1.67 | 0.804 |
| 50-54 | 0.68 | 0.35 | — | 1.31 | 0.247 |  | 0.99 | 0.49 | — | 1.98 | 0.977 |  | 0.86 | 0.47 | — | 1.58 | 0.633 |
| 55-57 | 1.00 |  |  |  |  |  | 1.00 |  |  |  |  |  | 1.00 |  |  |  |  |
| Workplace |  |  |  |  |  |  |  |  |  |  |  |  |  |  |  |  |  |
| Large cities | 1.00 |  |  |  |  |  | 1.00 |  |  |  |  |  | 1.00 |  |  |  |  |
| Small cities | 0.93 | 0.70 | — | 1.24 | 0.613 |  | 1.03 | 0.79 | — | 1.33 | 0.845 |  | 0.72 | 0.56 | — | 0.94 | 0.016 |
| Towns or villages | 1.02 | 0.57 | — | 1.85 | 0.938 |  | 1.01 | 0.57 | — | 1.78 | 0.978 |  | 0.46 | 0.22 | — | 0.98 | 0.044 |
| Number of board certifications |  |  |  |  |  |  |  |  |  |  |  |  |  |  |  |  |  |
| 0 | 1.00 |  |  |  |  |  | 1.00 |  |  |  |  |  | 1.00 |  |  |  |  |
| 1 | 0.93 | 0.65 | — | 1.35 | 0.714 |  | 0.82 | 0.59 | — | 1.15 | 0.252 |  | 0.90 | 0.67 | — | 1.21 | 0.497 |
| 2 or more | 0.84 | 0.52 | — | 1.35 | 0.468 |  | 0.57 | 0.36 | — | 0.91 | 0.018 |  | 0.85 | 0.57 | — | 1.26 | 0.406 |
| Type of work |  |  |  |  |  |  |  |  |  |  |  |  |  |  |  |  |  |
| Hospital/ clinic founder or director | 0.17 | 0.07 | — | 0.40 | <0.001 |  | 0.08 | 0.03 | — | 0.27 | <0.001 |  | 0.10 | 0.04 | — | 0.29 | <0.001 |
| Hospital staff | 1.00 |  |  |  |  |  | 1.00 |  |  |  |  |  | 1.00 |  |  |  |  |
| Clinic staff | 2.01 | 1.35 | — | 3.00 | 0.001 |  | 2.03 | 1.42 | — | 2.89 | <0.001 |  | 1.59 | 1.10 | — | 2.29 | 0.013 |
| Medical school* | 1.34 | 0.97 | — | 1.83 | 0.073 |  | 1.19 | 0.88 | — | 1.60 | 0.260 |  | 0.93 | 0.69 | — | 1.24 | 0.615 |
| Specialty |  |  |  |  |  |  |  |  |  |  |  |  |  |  |  |  |  |
| Internal medicine | 1.00 |  |  |  |  |  | 1.00 |  |  |  |  |  | 1.00 |  |  |  |  |
| Surgery | 0.70 | 0.45 | — | 1.11 | 0.128 |  | 0.67 | 0.44 | — | 1.01 | 0.057 |  | 0.80 | 0.55 | — | 1.16 | 0.235 |
| Pediatrics | 1.53 | 0.90 | — | 2.61 | 0.114 |  | 1.44 | 0.89 | — | 2.35 | 0.141 |  | 1.53 | 0.97 | — | 2.42 | 0.068 |
| Obstetrics/gynecology | 0.33 | 0.10 | — | 1.06 | 0.063 |  | 0.61 | 0.28 | — | 1.33 | 0.216 |  | 0.98 | 0.53 | — | 1.81 | 0.949 |
| Psychiatry/psychosomatic medicine | 4.24 | 2.84 | — | 6.33 | <0.001 |  | 3.21 | 2.22 | — | 4.64 | <0.001 |  | 3.00 | 2.08 | — | 4.33 | <0.001 |
| Other specialties | 0.88 | 0.59 | — | 1.30 | 0.519 |  | 0.63 | 0.43 | — | 0.93 | 0.020 |  | 0.65 | 0.45 | — | 0.94 | 0.023 |
| Junior resident | 0.98 | 0.53 | — | 1.79 | 0.938 |  | 1.13 | 0.65 | — | 1.98 | 0.658 |  | 0.88 | 0.47 | — | 1.65 | 0.699 |

*Clinical faculty members, clinical staff, or PhD students (clinical students).
